# Synthesis, Characterization, and Reactivity of Functionalized Trinuclear Iron–Sulfur Clusters – A New Class of Bioinspired Hydrogenase Models

**DOI:** 10.1002/ejic.201500574

**Published:** 2015-08-07

**Authors:** Manuel Kaiser, Günther Knör

**Affiliations:** [a] Institute of Inorganic Chemistry, Johannes Kepler University Linz (JKU), Altenbergerstr. 69, 4040 Linz, Austria, http://www.anorganik.jku.at

**Keywords:** Iron, Cluster compounds, ­Carbonyl ligands, Bioinspired catalysis, Enzyme models, Hydrogenase

## Abstract

The air- and moisture-stable iron–sulfur carbonyl clusters Fe_3_S_2_(CO)_7_(dppm) (**1**) and Fe_3_S_2_(CO)_7_(dppf) (**2**) carrying the bisphosphine ligands bis(diphenylphosphanyl)methane (dppm) and 1,1′-bis(diphenylphosphanyl)ferrocene (dppf) were prepared and fully characterized. Two alternative synthetic routes based on different thionation reactions of triiron dodecacarbonyl were tested. The molecular structures of the methylene-bridged compound **1** and the ferrocene-functionalized derivative **2** were determined by single-crystal X-ray diffraction. The catalytic reactivity of the trinuclear iron–sulfur cluster core for proton reduction in solution at low overpotential was demonstrated. These deeply colored bisphosphine-bridged sulfur-capped iron carbonyl systems are discussed as promising candidates for the development of new bioinspired model compounds of iron-based hydrogenases.

## Introduction

The sustainable production of energy-rich molecules such as hydrogen powered by renewable energy is considered as an attractive future alternative to fossil-fuel consumption.^1^ In this context, the search for catalytic systems capable of splitting water molecules into hydrogen and oxygen or peroxides [Equation (1)] has become a vibrant field of chemical research.^2^

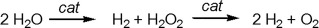
(1)

Although platinum-group metals still play a dominant role as the most efficient catalysts used today for technical hydrogen production, considerable efforts are currently focused on the replacement of the required multielectron-transfer reactivity of these systems with environmentally benign and earth-abundant metals. Important examples of nonprecious metal catalysts competent for hydride formation and proton reduction include first-row transition element complexes of iron,^3^ cobalt,^4^ and nickel^5^ and, more recently, also derivatives of main-group elements such as tin complexes carrying noninnocent ligands.^6^

In natural systems, the uptake and release of H_2_ is catalyzed by hydrogenases,^7^ a family of highly efficient metalloenzymes characterized by organometallic reaction centers with low-valent iron or nickel–iron sites as their common functional subunits. Strong-field ligands such as CO or cyanide are present in these enzymes and can apparently serve to stabilize the catalytic metal centers in their low-spin state and modify their electronic structure for optimized substrate interactions.^8^

Over recent decades, a broad range of structural and functional analogues of the active sites of hydrogenases have been studied. In particular, the development of mono- or dinuclear iron carbonyl complexes as biomimetic model compounds for [Fe]- and [FeFe]-hydrogenases has been described.^7^,^9^ Much less attention has been devoted to the design and investigation of bioinspired hydrogenase models involving multinuclear carbonyl complexes with more than two iron centers in their catalytic cores.^10^

**Scheme 1:**
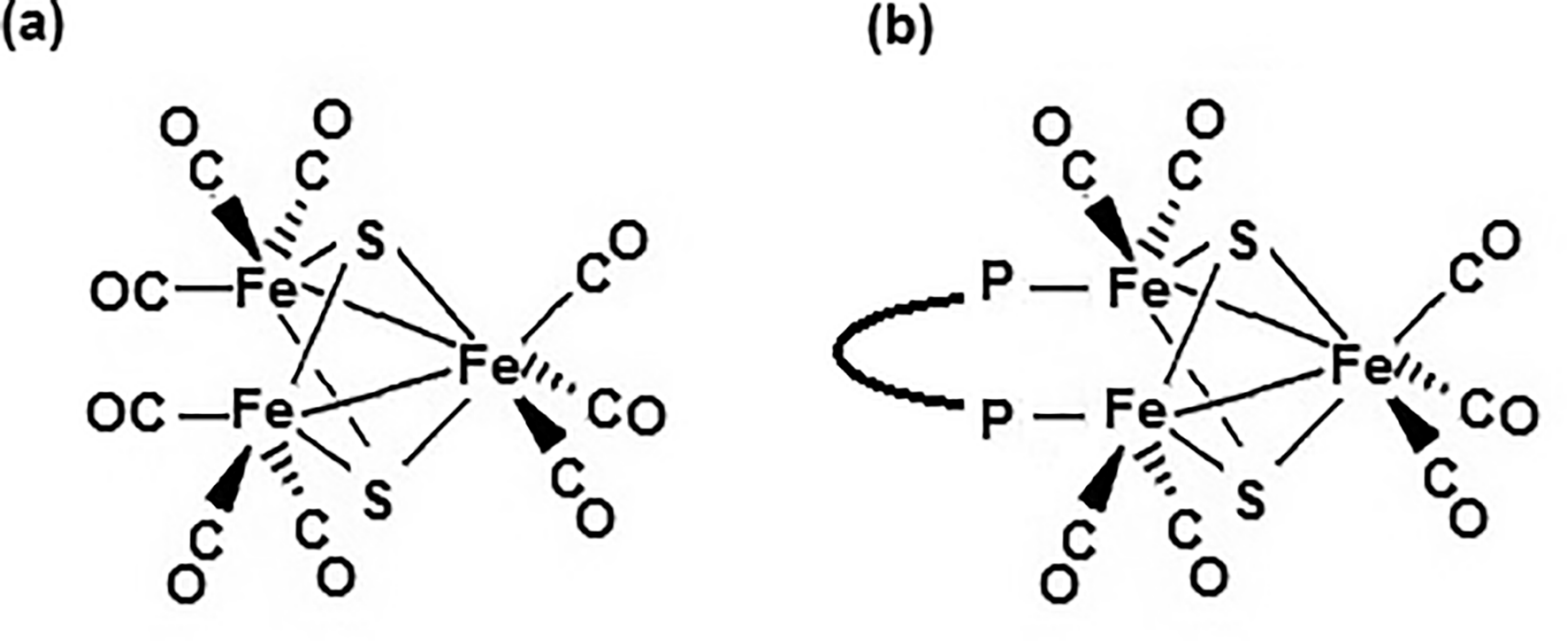
Structures of (a) the archetypical sulfur-capped triiron carbonyl cluster motif^11^ and (b) the bisphosphine derivatives studied in the present work.

Here, we present our results on the synthesis, characterization, and catalytic reactivity of trinuclear iron–sulfur clusters substituted with different bisphosphine ligands as a new class of robust artificial hydrogenases. Although the trinuclear iron carbonyl parent system Fe_3_(μ_3_-S)_2_(CO)_9_ has been known as an air- and water-stable compound for a long time,^11^,^12^ this family of functionalized iron carbonyl complexes (Scheme 1) has not yet been considered as potential catalysts for bioinspired hydrogen generation.

We decided to explore this possibility and started to study several modified Fe_3_(μ_3_-S)_2_(CO)_9_ clusters, in which two of the CO ligands are substituted by a bidentate phosphine subunit P–P (Scheme 1, b). In the present work, our data for the iron–sulfur clusters Fe_3_S_2_(CO)_7_(P–P) with P–P = bis(diphenylphosphanyl)methane (dppm) and 1,1′-bis(diphenylphosphanyl)ferrocene (dppf) are reported. The methylene-bridged derivative Fe_3_S_2_(CO)_7_(dppm) (**1**), which can be considered as the most simple representative of this class of compounds, was selected to probe the catalytic proton reduction activity of such cluster systems for the first time. On the other hand, the ferrocene-functionalized compound Fe_3_S_2_(CO)_7_(dppf) (**2**) was chosen as a potential second-generation catalyst carrying an additional redox mediator for multistep electron-transfer processes.^13^ Such an approach coupling substrate turnover at the low-valent cluster site with reversible electron transfer from a redox-active cofactor has already been proven to be advantageous for the construction of hydrogenase models based on a dinuclear iron core.^14^ Moreover, the additional ferrocene (Fc) subunit of Fe_3_S_2_(CO)_7_(dppf) will probably be able to serve as an intramolecular electron donor in photocatalytic systems for the reduction of protons to hydrogen.^15^

## Results and Discussion

**Scheme 2:**
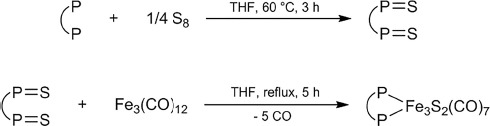
Synthesis of the iron–sulfur clusters **1** (P–P = dppm) and **2** (P–P = dppf) from triiron dodecacarbonyl and phosphine sulfide derivatives.

The air- and moisture-stable trinuclear iron–sulfur clusters **1** and **2** were prepared from the iron carbonyl precursor Fe_3_(CO)_12_. Two successful synthetic routes were established, and the routes rely on a different source of the capping sulfur atoms. First attempts were made according to the reaction sequence shown in Scheme 2, in which the bisphosphine sulfide derivatives dppmS_2_ and dppfS_2_ with P=S bonds acted as the thionation agents.

The chosen bisphosphine ligands were treated with elemental sulfur under a nitrogen atmosphere in dry tetrahydrofuran (THF) according to the literature method for dppmS_2_.^16^ The addition of Fe_3_(CO)_12_ under the same conditions afforded the deeply colored Fe_3_S_2_(CO)_7_(P–P) complexes as analytically pure crystalline materials after purification by column chromatography.

**Scheme 3:**
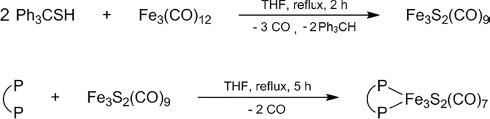
Synthesis of the iron–sulfur clusters **1** and **2** from triiron dodecacarbonyl, triphenylmethanethiol, and different bisphosphines (P–P = dppm, dppf).

In an alternative approach, these trinuclear iron–sulfur clusters were also obtained by using triphenylmethanethiol as a sulfur source in dry THF under nitrogen, as summarized in Scheme 3.

The isolation of the Fe_3_(μ_3_-S)_2_(CO)_9_ cluster formed in the first step of this reaction sequence was not required, and the corresponding phosphine ligand dppm or dppf was added after ca. 2 h of reaction time as soon as the formation of a metal mirror became noticeable.^17^ The isolated Fe_3_S_2_(CO)_7_(P–P) products were characterized by various spectroscopic methods after silica gel column chromatography.

The bidentate coordination mode of the bisphosphine ligands P–P was confirmed by the presence of a sharp singlet ^31^P NMR spectroscopy signal in CDCl_3_ solution, which occurs at *δ* = 75.4 ppm for the dppm complex and at *δ* = 68.4 ppm for the dppf derivative. These results are also consistent with a diamagnetic closed-shell character of the cluster compounds. The phosphine ligands strongly σ-donate electron density along the P–Fe bonds, which causes a more electron-rich iron core. At the same time this leads to a significant deshielding of the phosphorus atoms, and a characteristic downfield shift of the ^31^P resonance signals defined as Δ*δ* = (*δ*_complex_ – *δ*_free ligand_)^18^ occurs upon coordination. Although the chelation shifts are typically expected to be larger for the ferrocene-bridged bisphosphine ligand dppf than for the corresponding dppm complexes,^19^ the observed Δ*δ* values are 97.2 ppm for **1** and 85.4 ppm for **2**.

**Figure 1:**
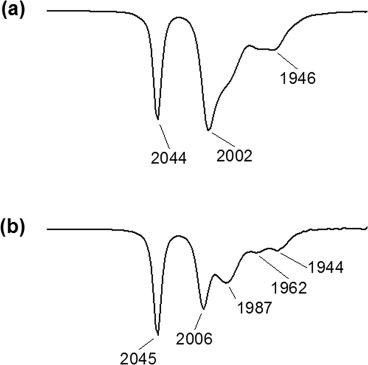
Solution FTIR spectra of the iron–sulfur clusters (a) **1** and (b) **2** in the carbonyl stretching vibration region (298 K, CH_2_Cl_2_).

The FTIR spectra of the triiron clusters were measured both in solution and in the solid phase. Notably, the KBr pellet data of **2** were characterized by a broadened and almost featureless peak pattern (see the Supporting Information), which sometimes occurs for this class of compounds in the solid state.^20^ Well-resolved spectra could be obtained for both clusters only in the liquid phase. The infrared spectrum of the iron–sulfur complex **1** in CH_2_Cl_2_ solution displays a set of three main carbonyl band maxima and two additional shoulders in the 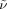 = 1900–2100 cm^–1^ region for ν(CO) stretching vibration (Figure1). A quite similar FTIR pattern is observed for **2**; however, the two additional ν(CO) shoulders at 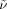 = 1987 and 1962 cm^–1^ are better resolved and more intense.

The energetic position of the carbonyl stretching vibrations also confirms the electron-rich character of the trinuclear iron core of both metal–sulfur clusters. Although the unsubstituted parent compound Fe_3_S_2_(CO)_9_ exhibits the highest frequency FTIR signal at 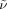 = 2064 cm^–1^ in solution,^17^ the corresponding carbonyl peaks are redshifted by Δν(CO) ≈ 20 cm^–1^ for the bisphosphine-functionalized Fe_3_S_2_(CO)_7_(P–P) derivatives (Figure1). This is consistent with an increased electron density of the iron d orbitals involved in π-backbonding to the attached carbon monoxide ligands. Clearly, the degree of π-back-donation from the iron core of the carbonyl clusters **1** and **2** is more pronounced than that typically observed for hydrogenase models based on a dinuclear iron core, for which the corresponding high-frequency CO-stretching signals are expected in the 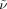 = 2070–2080 cm^–1^ range.^9^ For these latter systems, a linear correlation between the redshift Δν(CO) observed upon partial reduction and the spin-density population at the Fe–Fe core has been reported recently.^21^ An electron-rich situation with spin-density localized at the Fe cluster moiety is considered to be a crucial prerequisite for photo- and electrocatalytic hydrogen production. Compared to the situation in dinuclear hydrogenase models, the observed vibrational frequency shift of almost 40 cm^–1^ in their oxidized resting state should make complexes such as **1** and **2** promising candidates for studies of electron-transfer-triggered proton to hydrogen reduction in solution.

**Figure 2:**
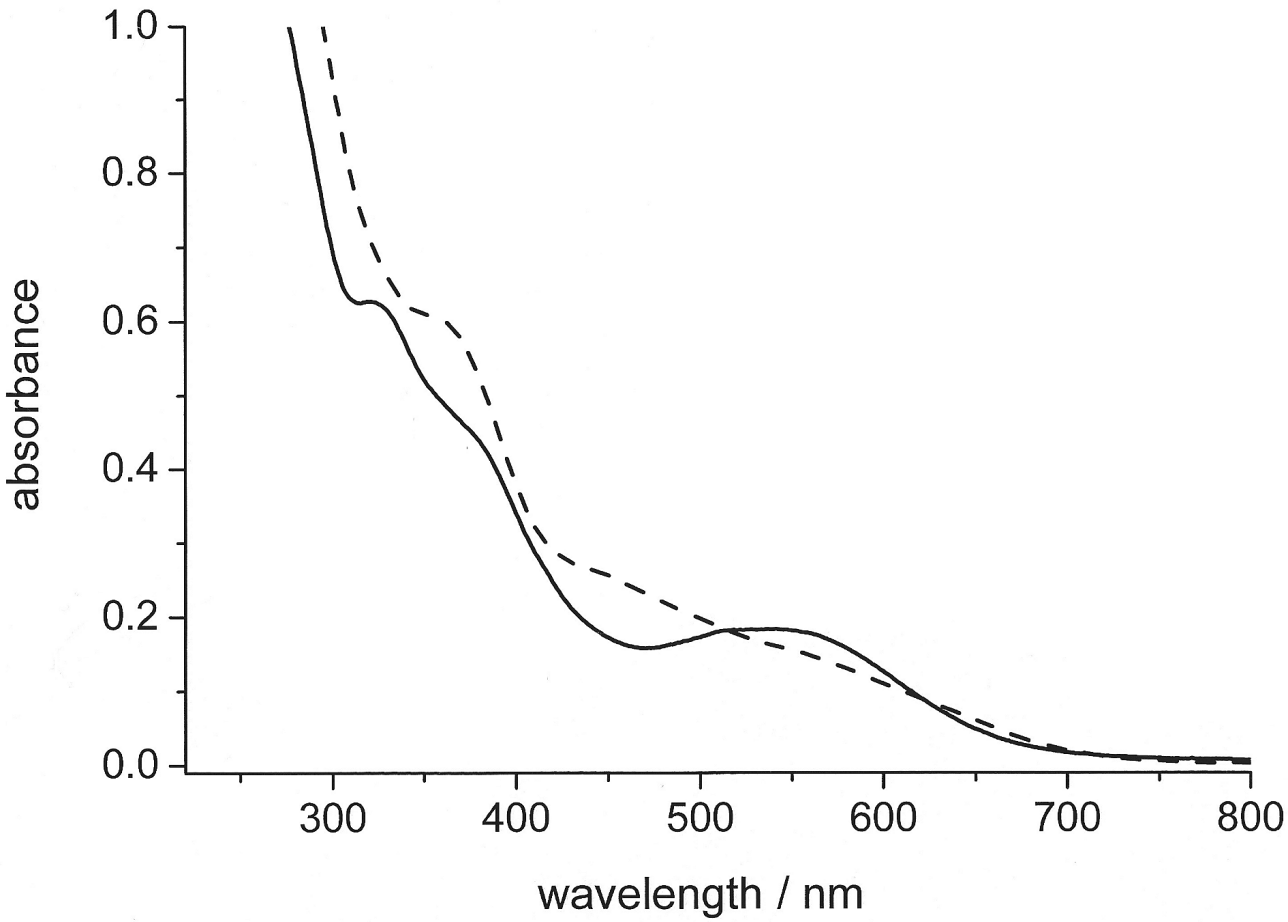
Electronic absorption spectra of 5 × 10^–5^ M **1** (^____^) and 6 × 10^–5^ M **2** (- - -) in CH_2_Cl_2_ solution (298 K, 1 cm cell).

The UV/Vis spectra of the iron–sulfur carbonyl clusters **1** and **2** are shown in Figure2. Both compounds are intensely colored, almost black crystalline materials, which dissolve readily in organic solvents such as dichloromethane, acetone, or methanol to form dark red (**1**) or brownish (**2**) solutions. No deviations from the Lambert–Beer law up to a concentration of 10^–4^ M and no significant influence of solvent polarity changes on the spectral characteristics were observed; this suggests a delocalized ground-state electronic structure. This lack of solvatochromism is not surprising, as in contrast to the situation in compounds derived from dinuclear Fe_2_S_2_(CO)_6_ containing an iron-bridging disulfide ligand, no low-lying σ*(S–S) acceptor orbitals are available in the Fe_3_S_2_(CO)_9_ derivatives for dσ* metal-to-ligand charge-transfer (MLCT) transitions.^22^ The chromophoric bands in the visible spectral region most probably arise from allowed electronic transitions within the σ-bonded Fe_3_ triangle of the iron cluster core^23^ and display a rather high intensity with molar extinction coefficients in the range 3000–4000 M^–1^ cm^–1^ (Figure2). Although the dppm derivative **1** is characterized by well-defined absorption maxima at *λ* = 320, 380, and 550 nm, the spectrum of the dppf-bridged cluster **2** is somewhat less resolved owing to the presence of additional absorption bands at *λ* ≈ 350 and 450 nm (Figure2). These latter features almost coincide with the peak maxima of the free ferrocene chromophore, which occur at *λ* = 330 and 440 nm.^24^ However, in addition to a moderate bathochromic shift of these peaks for **2**, the intensity of the electric-dipole-forbidden d–d absorptions of the ferrocene subunit is increased significantly by a factor of approximately ten, which indicates a certain degree of mixing of the electronic wave functions and this decreases the intensity of the dipole-allowed cluster core transitions.

**Figure 3:**
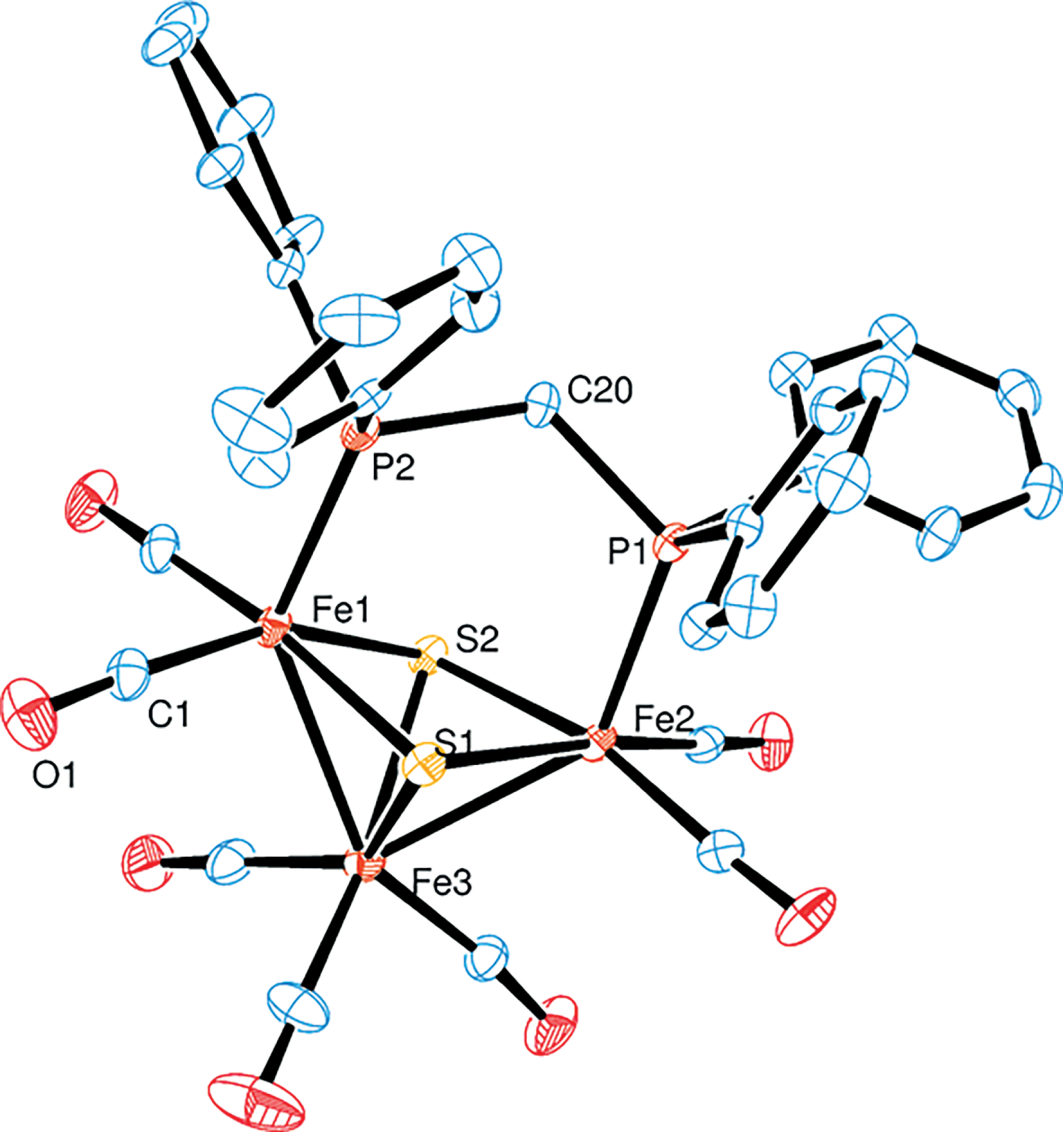
Molecular structure of the methylene-bridged iron–sulfur cluster **1** (ORTEP; displacement ellipsoids at the 50 % probability level; H atoms are omitted for clarity).

**Figure 4:**
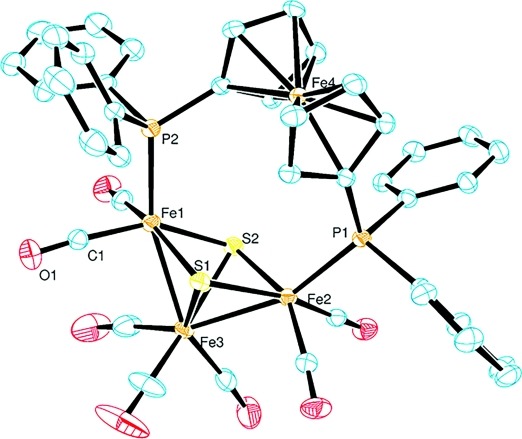
Molecular structure of the ferrocene-bridged iron–sulfur cluster **2** (ORTEP; displacement ellipsoids at the 50 % probability level; H atoms are omitted for clarity).

To further characterize the bonding properties of the trinuclear iron–sulfur clusters, the molecular structures of the Fe_3_S_2_(CO)_7_(P–P) derivatives were studied by X-ray diffraction. Single crystals of **1** (Figure3) were obtained by the slow gas-phase diffusion of diethyl ether into a solution of the compound in CH_2_Cl_2_. For **2** (Figure4), suitable single crystals were grown by evaporation of the solvent after purification by column chromatography. The compound was dissolved in acetone, and evaporation in air gave crystals with cyclohexane in the crystal lattice.

The structures of both compounds are characterized by a triangular arrangement of the iron centers, and two μ_3_-capping sulfur atoms form a square-pyramidal *nido*-type Fe_3_S_2_ core with two Fe–Fe bonds in accordance with polyhedral skeletal electron pair theory.^12^ As expected from the IR data, all carbonyl ligands are bound in a terminal fashion. Selected structural data for **1** and **2** are summarized in Table1. The crystallographic refinement data can be found in Table2.

**Table 1:** Selected bond lengths [Å] and angles [°] of 1 and 2.

Bond lengths	1	2
Fe3–Fe1	2.607(1)	2.568(6)
Fe3–Fe2	2.615(1)	2.576(6)
S1–S2	2.856(2)	2.909(6)
Fe3–S1	2.256(2)	2.276(9)
Fe3–S2	2.265(2)	2.302(9)
Fe1–S1	2.239(2)	2.250(9)
Fe1–S2	2.240(2)	2.262(1)
Fe2–S1	2.236(2)	2.256(9)
Fe2–S2	2.249(2)	2.260(9)
Fe1–P2	2.212(2)	2.234(9)
Fe2–P1	2.185(2)	2.230(9)
Bond angles		
Fe1–Fe3–Fe2	80.3(4)	85.4(2)
Fe1–S1–Fe2	97.6(6)	101.5(4)
Fe1–S2–Fe2	97.2(6)	101.0(4)
S1–Fe1–S2	81.0(6)	78.6(3)
S1–Fe2–S2	80.9(6)	78.5(3)
S1–Fe3–S2	80.1(6)	77.2(3)
P2–Fe1–Fe3	137.3(6)	159.4(3)
P1–Fe2–Fe3	137.5(5)	159.4(3)

**Table 2:** Crystal data, data collection, and structure refinement for 1 and 2.

	1	2
Formula	C_32_H_22_Fe_3_O_7_P_2_S_2_	C_41_H_28_Fe_4_O_7_P_2_S_2_**·**2(C_6_H_12_)
*M*_W_ [g mol^–1^]	819.16	1150.41
Crystal size [mm]	0.44 × 0.26 × 0.14	0.45 × 0.42 × 0.38
Crystal system	triclinic	triclinic
Space group	*P* 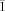	*P* 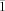
*a* [Å]	11.083(1)	11.799(9)
*b* [Å]	11.571(1)	12.948(1)
*c* [Å]	14.269(2)	17.383(2)
*α* [°]	100.884(4)	77.360(3)
*β* [°]	95.536(4)	86.629(3)
*γ* [°]	112.728(3)	82.843(3)
*V* [Å^3^]	1628.2(3)	2569.7(4)
*ρ*_calcd._ [g cm^–3^]	1.671	1.487
*Z*	2	2
*μ* [mm^–1^]	1.59	1.30
*T* [K]	210	298
*Θ* range [°]	2.2–24.4	2.2–26.7
*λ* [Å]	0.71073	0.71073
Reflections collected	32458	60713
Unique reflections	6275	11906
Observed reflections [*I* > 2σ(*I*)]	4327	7794
Parameters refined/restraints	415/0	613/0
Absorption correction	multiscan	multiscan
*T*_min_, *T*_max_	0.51, 0.81	0.38, 0.64
*σ*_fin_ (max/min) [e Å^–3^]	1.17/–1.53	0.42/–0.47
*R*_1_ [*I* ≥ 2σ(*I*)]	0.073	0.047
*wR*_2_	0.234	0.120

Although the ferrocene bridge present in **2** is much larger than the methylene bridge of the bisphosphine ligand bound in **1**, this difference is not very significant with respect to the iron–sulfur cluster core. Indeed, both structures show quite similar bond properties, except from the larger bite angle of the dppf ligand in **2**, as reflected by a considerable flattening of the phosphorus to Fe–Fe bond connection line (Table1). In addition, the angle of the σ-bonded Fe_3_ fragment of the ferrocenyl-bridged derivative is slightly widened by ca. 5°. Nevertheless, the iron–sulfur and metal–metal bond lengths of **1** and **2** are very similar to the values reported for the unsubstituted Fe_3_(μ_3_-S)_2_(CO)_9_ cluster.^25^ The two cyclopentadienyl rings of the ferrocene subunit in **2** are inclined by 3° towards each other and have a torsion of ca. 8°. Therefore, the rings are almost coplanar and eclipsed, which is close to the predicted equilibrium structure of the metallocene.^26^

**Figure 5:**
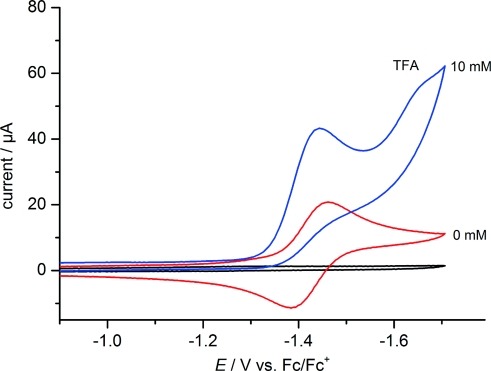
Cyclic voltammograms of the iron–sulfur cluster **1** (1.0 mM) in acetonitrile solution with and without the addition of 10 mM trifluoroacetic acid (TFA; 298 K, 0.1 M Bu_4_NPF_6_, glassy carbon working electrode, 100 mV s^–1^ scan rate).

The electrochemical properties of the dppm-functionalized triiron carbonyl cluster were studied by cyclic voltammetry. On the cathodic scan, the voltammogram of **1** in CH_2_Cl_2_ solution (1.5 mM, 0.1 M Bu_4_NPF_6_, 100 mV s^–1^ scan rate) displays a quasireversible first one-electron reduction wave at –1.60 V versus Fc^+^/Fc (see the Supporting Information for further details). In acetonitrile solution, the same complex shows a reversible one-electron reduction at –1.43 V (Figure5). The literature values reported for the Fe_3_S_2_(CO)_9_ parent system are –1.03 V and –0.94 V versus Fc^+^/Fc in CH_2_Cl_2_ and acetonitrile, respectively.^10^ From this comparison, it can be concluded that the reduction of the more-electron-rich Fe_3_S_2_(CO)_7_(dppm) derivative **1** requires a 500–600 mV more negative potential than that of the unmodified triiron cluster under similar conditions.

In the presence of a proton source such as trifluoroacetic acid (TFA), the first reduction wave of the iron–sulfur complex **1** becomes irreversible. The cathodic peak potential of this redox process is slightly shifted to more positive values and, at the same time, a catalytic peak current indicating hydrogen production is observed (Figure5).

**Figure 6:**
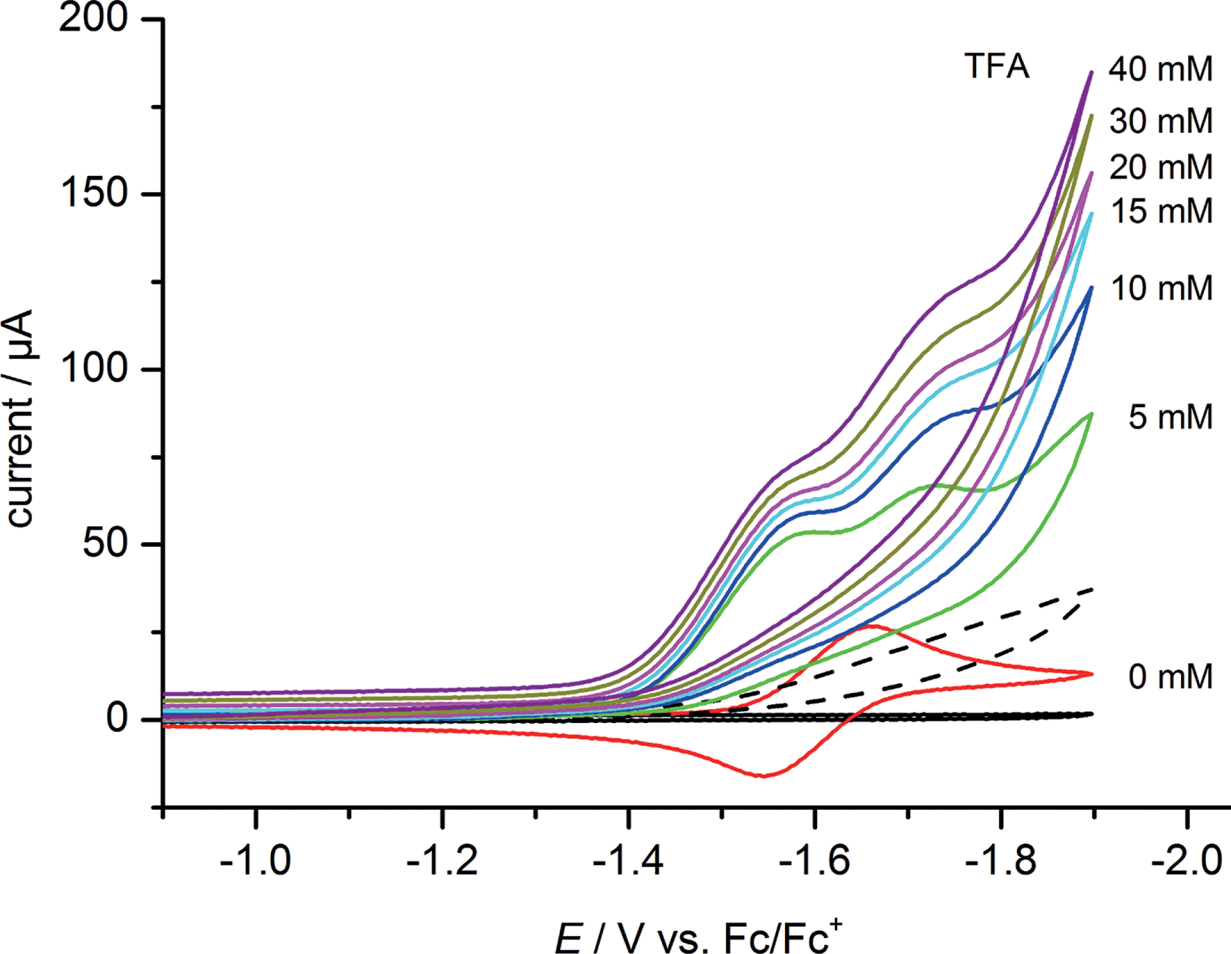
Cyclic voltammograms of the iron–sulfur cluster **1** (1.5 mM) in CH_2_Cl_2_ at increasing concentrations of TFA. The solvent baseline and the cyclic voltammogram of 5 mM TFA alone (- - -) are also shown (298 K, 0.1 M Bu_4_NPF_6_, glassy carbon working electrode, 100 mV s^–1^ scan rate).

A quite similar behavior is observed in CH_2_Cl_2_ containing TFA as a proton source. However, in addition to the small positive shift in the position of the first reduction wave, a second reduction process also appears here at a peak potential of ca. –1.7 V versus Fc^+^/Fc (Figure6). The half-peak potentials of the catalytic waves for the reduction of protons are –1.37 and –1.48 V versus Fc^+^/Fc in acetonitrile and CH_2_Cl_2_ solution, respectively. Initially, the catalytic peak currents *i*_cat_ increase with the amount of TFA added. At higher acid concentrations, this increase levels off, and the process becomes independent of the proton concentration, which can be interpreted in terms of a rate-limiting elimination of H_2_ under these conditions.^27^ From the limiting current in this acid-independent region, a turnover frequency (TOF) of 100 min^–1^ is obtained for the dppm-bridged iron cluster **1** in acetonitrile.^27^,^28^ From the operating potential of this catalyst and the p*K*_a_ value of TFA (12.65), the overpotential for hydrogen production can be estimated to be 540 mV by using the reported value of –0.260 V versus Fc^+^/Fc in acetonitrile for the solvated proton/dihydrogen couple (see the Supporting Information).^29^ At the half-peak potential of the catalytic wave (Figure5), H_2_ evolution by the hydrogenase model **1** in acetonitrile solution containing TFA occurs with a current density of ca. 400 μA cm^–2^. A limiting value of ca. 0.9 mA cm^–2^ is observed for a 1 mM solution of **1** containing an excess of acid; this value and the observed TOF value are in reasonable agreement with the theoretical performance expected.^30^

The positive shift in the position of the first reduction wave of **1** in the presence of TFA (Figure6) indicates that the protonation of the iron–sulfur cluster core already occurs at low acid concentration, which interestingly is not the case for the less electron-rich Fe_3_S_2_(CO)_9_ parent system.^10^ This behavior is also indicated by the UV/Vis spectral changes that occur upon the titration of **1** with TFA (see the Supporting Information). In particular, the electronic transitions in the visible spectral region related to the Fe–Fe σ-bonded cluster core are strongly affected by protonation. Such a reactivity should also cause a shift of the ν(CO) vibration frequencies to higher energies owing to a decreasing electron density of the iron d orbitals involved in π-backbonding to the attached carbonyl ligands. Indeed, the FTIR spectra of **1** in CH_2_Cl_2_ with increasing amounts of TFA added clearly provide evidence for a stepwise protonation process. The observed large blueshifts of the carbonyl stretching vibrations attributed to the formation of the mono- and diprotonated forms of **1** are Δν(CO) = 75 cm^–1^ for the first step and Δν(CO) = 100 cm^–1^ after the second step (see the Supporting Information for further details). These results are consistent with the generation of iron species with μ-bridging hydrido ligands.^9^ Therefore, the conversion of **1** to the mono- and diprotonated cationic complexes [Fe_3_S_2_(CO)_7_(dppm)(μ-H)]^+^ and [Fe_3_S_2_(CO)_7_(dppm)(μ-H)_2_]^2+^ is proposed to explain the spectroscopic results observed in the presence of TFA. Further studies by ^1^H NMR spectroscopy in the diagnostic high-field region^31^ to confirm this assumption are currently underway, as an alternative protonation at the sulfur centers cannot be excluded fully.^32^

In analogy to the mechanisms discussed for dinuclear iron-based hydrogenase model compounds,^7^,^9^ the subsequent electro- or photoreduction of these iron hydrido complexes formed in the presence of a moderately strong acid such as TFA is expected to trigger the release of H_2_ in a protic environment. In our case, this rate-limiting final step of the catalytic hydrogen production cycle should regenerate the Fe–Fe-bonded cluster core of the Fe_3_S_2_(CO)_7_(dppm) starting complex **1**. As the similarities in the energetic positions of the carbonyl group stretching vibrations (Figure1) suggest a closely related electronic structure of the σ-bonded triiron core, the same kind of reactivity should also be present with other derivatives of the bisphosphine-functionalized family of Fe_3_S_2_(CO)_7_(P–P) clusters such as **2**, for which an additional redox mediator is attached to the catalytic site. Further improvements of such bioinspired hydrogenase model systems could be expected by the inclusion of a proton relay subunit^14^ to accelerate the terminal step of hydrogen gas release from the reduced catalyst. However, such an additional functionalization was not within the scope of the present study.

## Conclusions

In the present work, we have introduced phosphine-modified trinuclear iron carbonyl clusters as new examples of bioinspired hydrogenase enzyme models. These air- and moisture-stable organometallic complexes are exceptionally electron-rich compounds, which can interact readily with protons in solution. Evidence for the stepwise formation of μ-hydrido species in the presence of trifluoroacetic acid was obtained. The iron hydrido intermediates formed are able to accelerate the release of H_2_ upon reduction in protic media, which makes them attractive candidates as nonprecious-metal-based multielectron transfer reagents for the electro- or photocatalytic generation of hydrogen as a renewable solar fuel. Therefore, more detailed investigations of this promising family of compounds including NMR spectroscopy, Mössbauer spectroscopy, photochemical reactivity, and ultrafast vibrational spectroscopy are currently underway.

## Experimental Section

**General Methods:** Reactions and manipulations of air- and moisture-sensitive compounds were performed under an atmosphere of dry nitrogen by using standard Schlenk techniques. All solvents and other reagents were commercially available and used as received. The NMR spectra were recorded with an Avance DRX 300 (300 MHz) spectrometer. The ^1^H and ^13^C shifts are reported in ppm relative to SiMe_4_ and were referenced internally to the residual signals of the deuterated solvent. The ^31^P shifts are reported in ppm relative to phosphoric acid. The UV/Vis spectra were recorded with a Cary 50 spectrophotometer. The infrared spectra were obtained with a Shimadzu IR-Affinity-1 spectrometer. Cyclic voltammograms were obtained with an Eco Autolab system by employing a standard three-electrode cell equipped with a BAS glassy carbon working electrode (*A* = 0.0707 cm^2^), a platinum wire counter electrode, and a silver/silver chloride pseudoreference electrode. 0.1 M Bu_4_NPF_6_ was used as the supporting electrolyte, and ferrocene was used as an internal standard for potential referencing. All measurements were accomplished under a nitrogen atmosphere at room temperature. Single-crystal structure analysis was performed with a Bruker Smart X2S diffractometer with graphite-monochromated Mo-*K*_α_ radiation (*λ* = 0.71073 Å). The structures were solved by direct methods (SHELXS-97) and refined by full-matrix least-squares techniques on *F*^2^ (SHELXL-97). The H atoms were calculated geometrically, and a riding model was applied during the refinement process. CCDC-1059276 (for **1**) and -CCDC-1059277 (for **2**) contain the supplementary crystallographic data for this paper. These data can be obtained free of charge from The Cambridge Crystallographic Data Centre via www.ccdc.cam.ac.uk/data_request/cif.

**Synthesis:** Bis(diphenylthiophosphinoyl)methane (dppmS_2_) was prepared according to a literature procedure.^16^ The reaction with 1,1′-bis(diphenylphosphanyl)ferrocene (dppf) as a starting material was performed analogously and resulted in dppfS_2_ as a yellowish powder in preparative yield. ^31^P NMR (121.5 MHz, CDCl_3_): *δ* = 40.7 (s).

**Fe_3_S_2_(CO)_7_(dppm), (1):** Fe_3_(CO)_12_ (344 mg, 0.68 mmol) and dppmS_2_ (300 mg, 0.67 mmol) were dissolved in THF (15 mL), and the mixture was heated under reflux for 5 h. The removal of the solvent and purification by column chromatography (CH_2_Cl_2_/cyclohexane 1:1) gave a very dark red, almost black microcrystalline powder in 39 % yield (192 mg, 0.26 mmol). ^31^P NMR (121.5 MHz, CDCl_3_): *δ* = 75.4 (s). IR [KBr pellet, ν(CO)]: 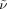 = 2041, 2004, 1979, 1954, 1937 cm^–1^.

**Fe_3_S_2_(CO)_7_(dppf), (2):** Fe_3_(CO)_12_ (500 mg, 1.09 mmol) and dppfS_2_ (675 mg, 0.99 mmol) were treated analogously to **1**. The reaction afforded an almost black microcrystalline powder in 8 % yield (32 mg, 0.03 mmol). ^31^P NMR (121.5 MHz, CDCl_3_): *δ* = 68.4 (s). IR [KBr pellet, ν(CO)]: 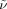 = 2062–1927 (very broad and flat signal) cm^–1^.

**Alternative Synthetic Route:** Fe_3_(CO)_12_ (200 mg, 0.40 mmol) and triphenylmethanethiol (220 mg, 0.79 mmol) were dissolved in THF (25 mL), and the mixture was heated under reflux for 2 h until a metal mirror was visible.^17^ The required bisphosphine ligand (dppm, 168 mg, 0.44 mmol or dppf, 242 mg, 0.44 mmol) was added, and the mixture was further heated under reflux for 5 h. The removal of the solvent and purification by column chromatography (CH_2_Cl_2_/cyclohexane 1:1) afforded the pure product in a lower yield (88 mg, 0.11 mmol, 27 % vs. 39 % for **1** and 5 % vs. 8 % for **2**).

## Supporting Information

As a service to our authors and readers, this journal provides supporting information supplied by the authors. Such materials are peer reviewed and may be re-organized for online delivery, but are not copy-edited or typeset. Technical support issues arising from supporting information (other than missing files) should be addressed to the authors.

miscellaneous_information
