# Supplementary material for: Synthesis, Characterization, and Reactivity of Functionalized Trinuclear Iron–Sulfur Clusters – A New Class of Bioinspired Hydrogenase Models
Source: Eur J Inorg Chem. 2015 Aug 7;2015(25):4199–206. doi: 10.1002/ejic.201500574 (PMC4612652; doi:10.1002/ejic.201500574)
Supplement: Supplementary file 1 [file ejic2015-4199-sd1.pdf]

Eur. J. Inorg. Chem. **2015** · ISSN 1099–0682

SUPPORTING INFORMATION

DOI: 10.1002/ejic.201500574
Title: Synthesis, Characterization, and Reactivity of Functionalized Trinuclear Iron–Sulfur Clusters – A New Class of Bioinspired Hydrogenase Models
Author(s): Manuel Kaiser, Günther Knör*

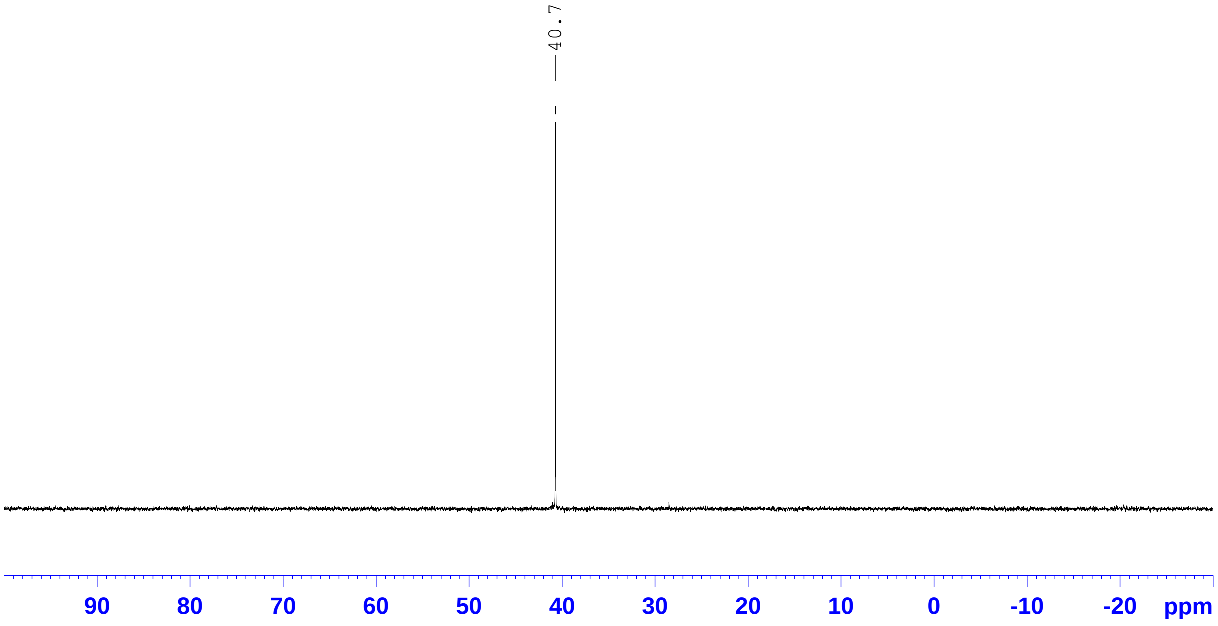

Figure S1. ^{31}P-NMR spectrum of 1,1'-Bis(diphenylthiophosphinoyl)methane (dppfS_2).

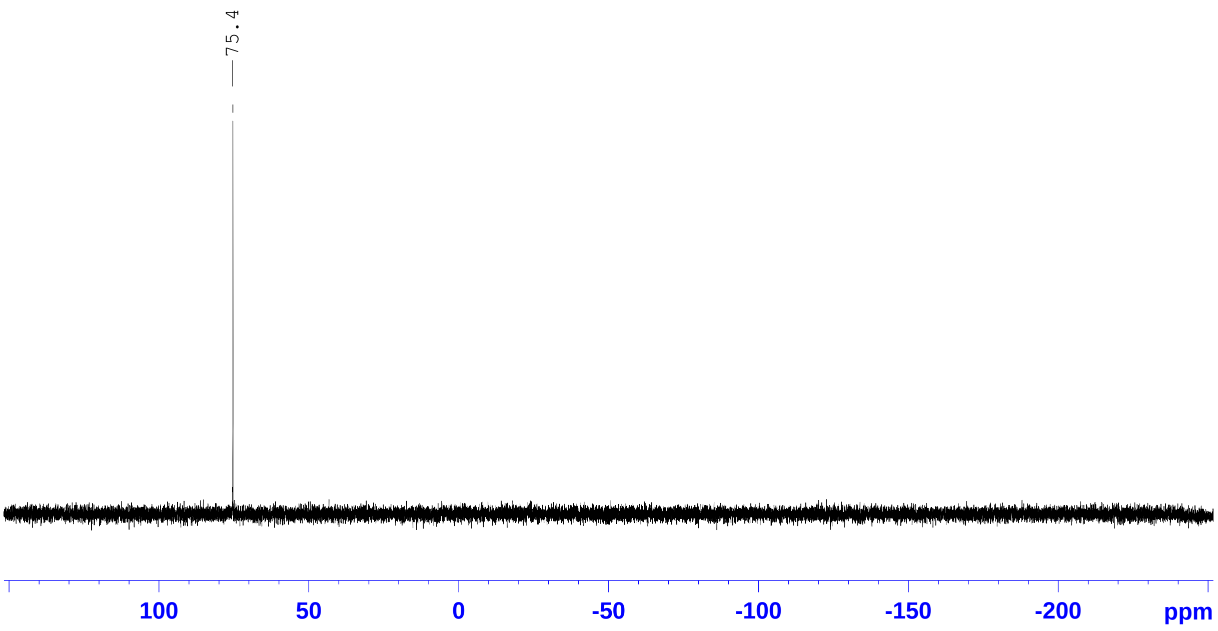

Figure S2. ^{31}P-NMR spectrum of $Fe_3S_2(CO)_7$(dppm), **1**.

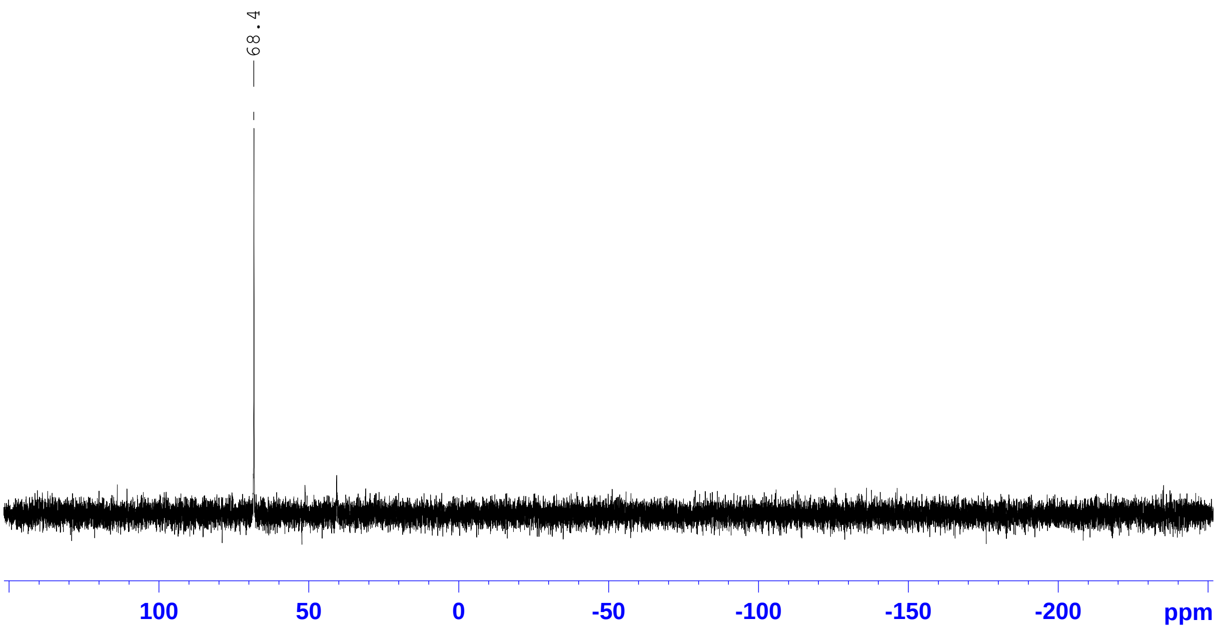

Figure S3. ^{31}P-NMR spectrum of $Fe_3S_2(CO)_7$(dppf), **2**.

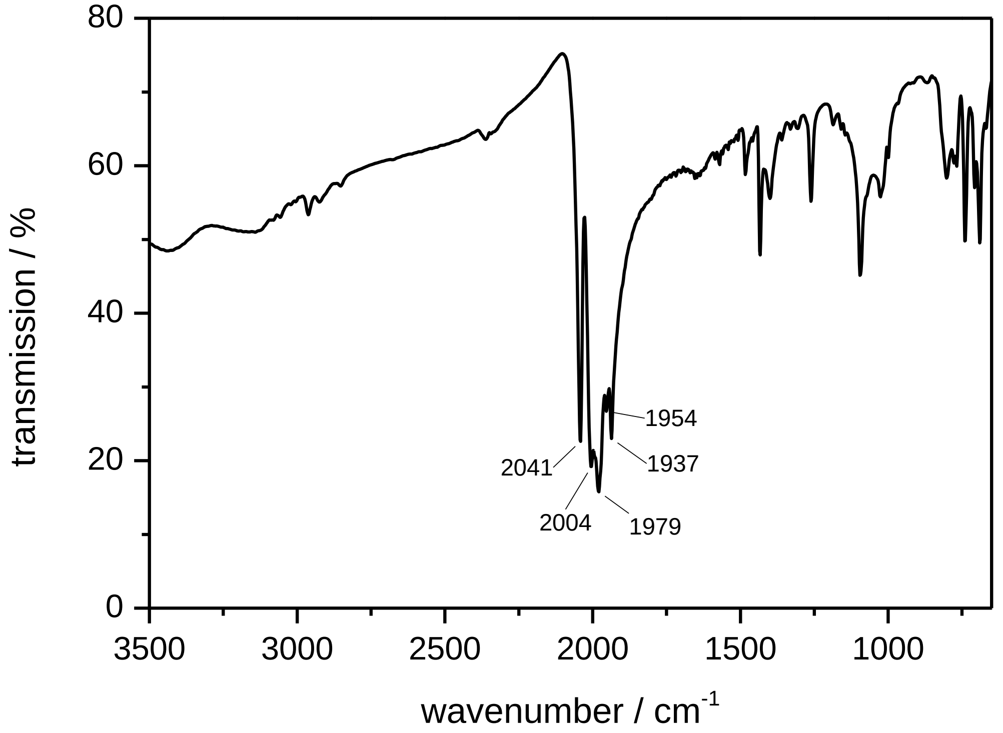

Figure S4. FTIR spectrum of $Fe_3S_2(CO)_7$(dppm), **1**, KBr-pellet.

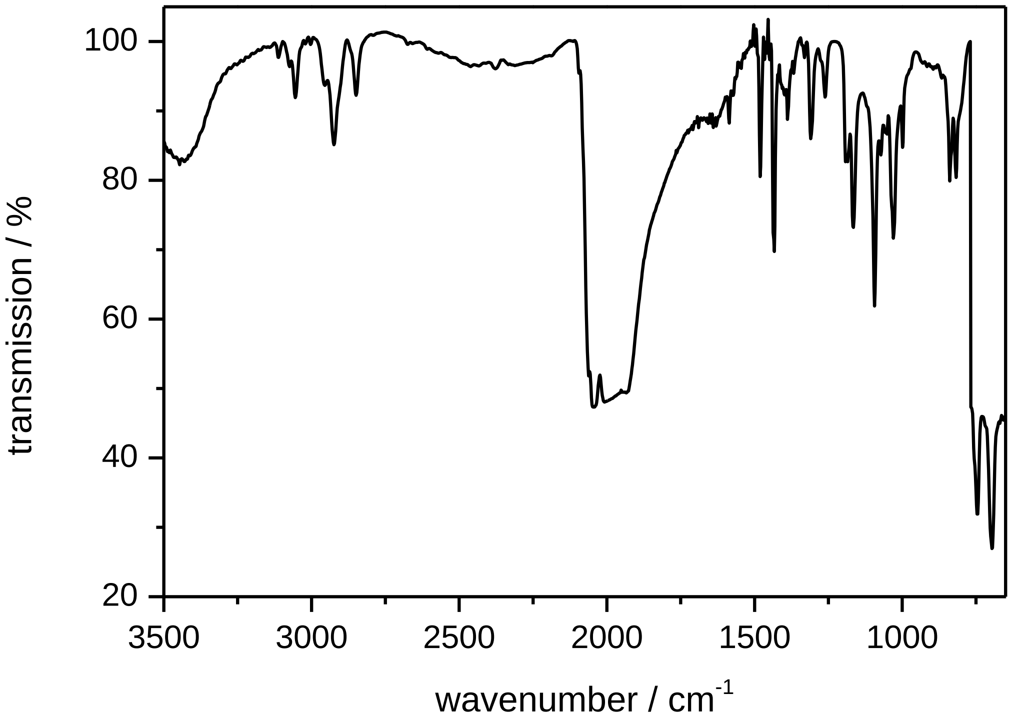

Figure S5. FTIR spectrum of $Fe_3S_2(CO)_7$(dppf), **2**, KBr-pellet.

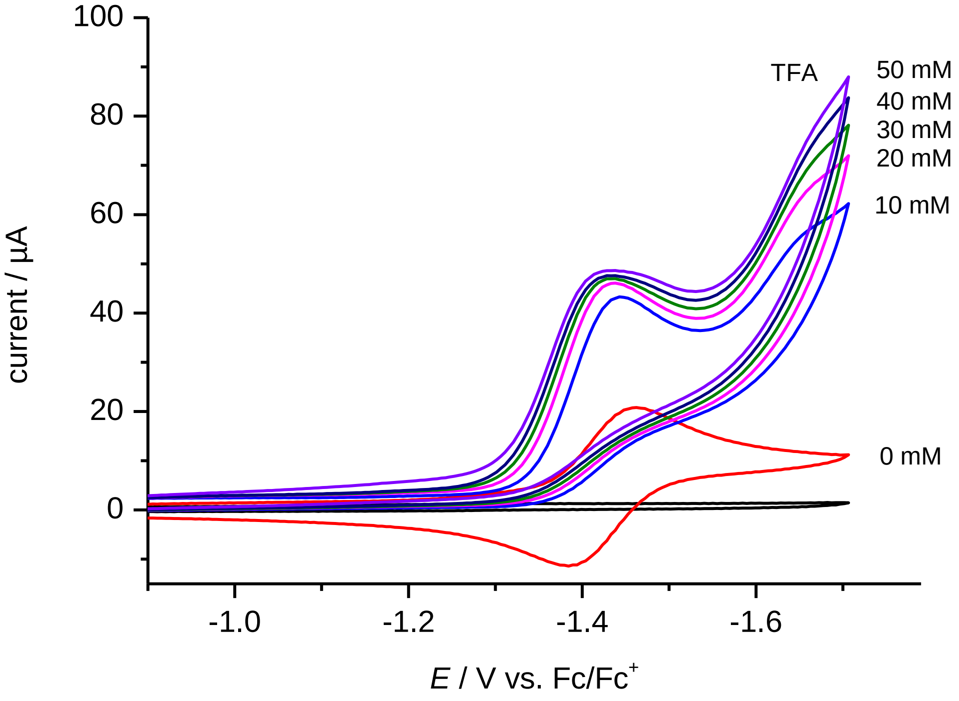

Figure S6. Cyclic voltammograms of **1** (1.0 mM) in acetonitrile with additions of TFA (298K, 0.1M Bu_4NPF_6, glassy carbon working electrode, scan rate 100mV/s).

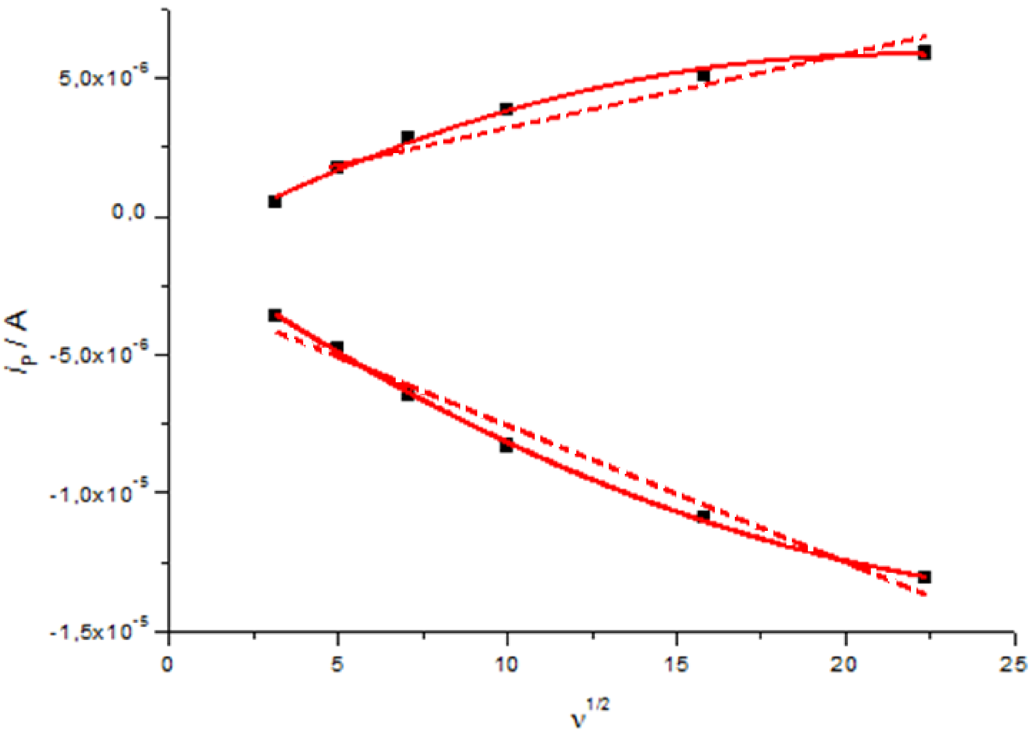

Figure S7. Nonlinear scan rate dependency of cathodic and anodic peak currents for the first reduction process of **1** in dichloromethane solution (values shown for 10, 20, 50, 100, 250, and 500 mV/s).

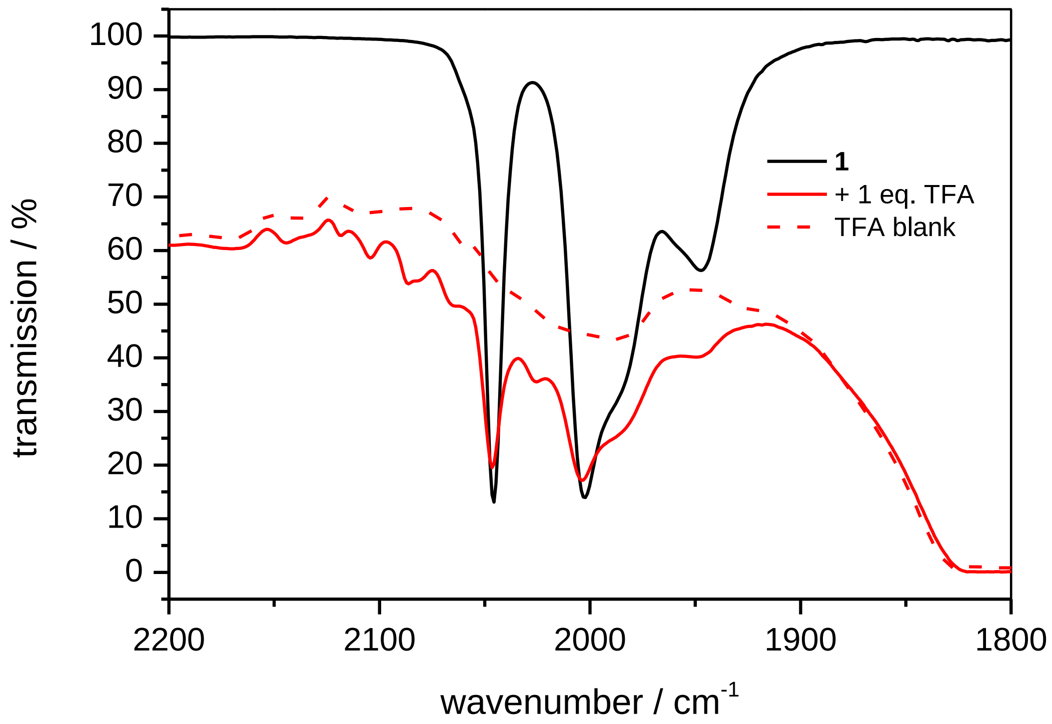

Figure S8. FTIR solution spectra of **1** in dichlorometane with the addition of TFA

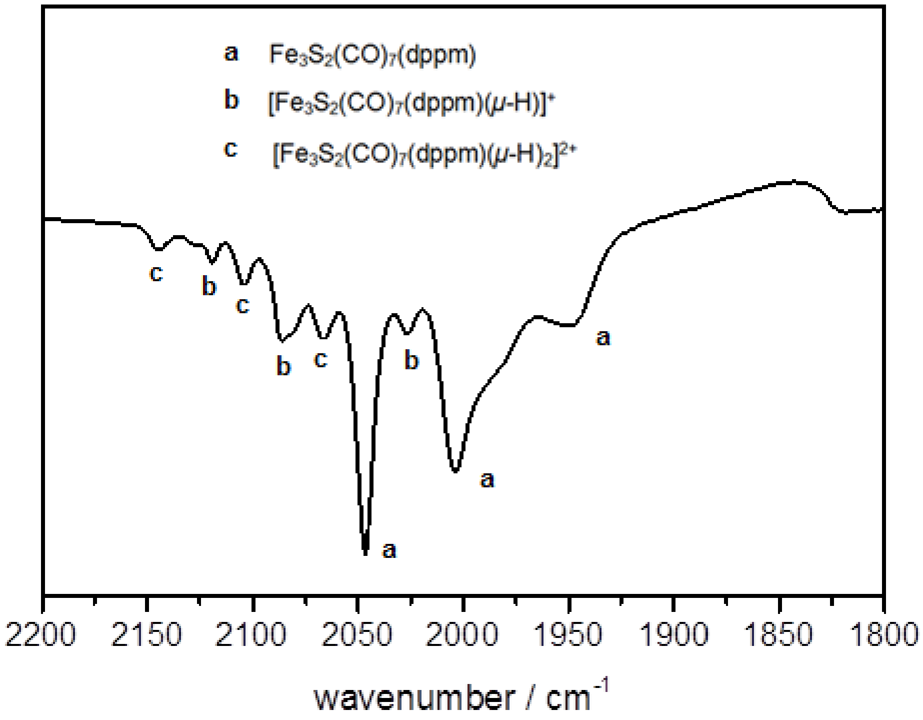

Figure S9. Differential spectrum of Fig. S8. The spectrum of **1** + 1 eq. TFA was substracted by the TFA spectrum.

Table S1. Main carbonyl stretching vibrations of **1**, as well as the tentatively assigned blue-shifted signals of mono- and di-protonated forms of **1** observed upon TFA addition in dichloromethane solution.

Abbreviation (see Fig. S9)	Compound	ν(CO) / cm^{-1}	ν(CO) / cm^{-1}	ν(CO) / cm^{-1}
**a:** (**1**)	$Fe_3S_2(CO)_7(dppm)$	2044	2002	1946
**b:** (**1**) + H^+	$[Fe_3S_2(CO)_7(dppm)(\mu\text{-}H)]^+$	2118*	2087	2025*
**c:** (**1**) + 2 H^+	$[Fe_3S_2(CO)_7(dppm)(\mu\text{-}H)_2]^{2+}$	2144	2103	2064

* signals lost at higher proton concentration

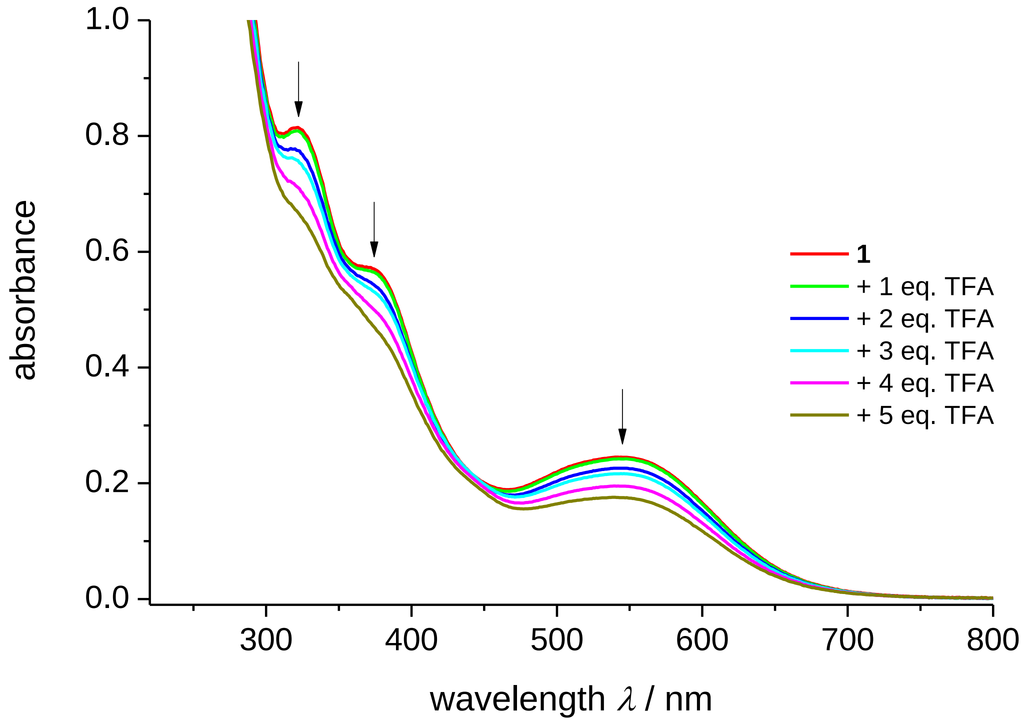

Figure S10. UV-vis absorption spectral changes of **1** in dichloromethane occurring with addition of TFA.

Further details on electrochemical experiments:

Peak potential separations ΔE_p and ratios of anodic and cathodic peak currents observed in the cyclovoltammetric experiments were used as a diagnostic criteria for distinguishing electrochemically reversible, irreversible and quasireversible processes [J. Heinze, *Angew. Chem.* **96** (1984) 893]. The condition $\Delta E_p = 59/n$ was applied for the determination of n-values of the corresponding reversible electron transfer steps.

Overpotential of catalytic proton reduction by $Fe_3S_2(CO)_7$dppm using TFA in acetonitrile at 298K:

$E(HA) = E(H^+) - 0.059\ pK_a$ with $E(H^+) = -0.260$ V [G. A. N. Felton, R. S. Glass, D. L. Lichtenberger, D. H. Evans, *Inorg. Chem.* **45** (2006) 9181] and **pK_a of TFA = 12.65** in acetonitrile [F. Eckert, I. Leito, I. Kaljurand, A. Kütt, A. Klamt, M. Diedenhofen, *J. Comput. Chem.* **30** (2009) 799] were used for calculating the **standard potential of TFA in acetonitrile: -1.006 V vs. Fc+/Fc**.

Determination of overpotentials for homoconjugation-free acids was applied for TFA according to recommended literature procedures: [V. Fourmond, P.-A. Jacques, M. Fontecave, V. Artero, *Inorg. Chem.* **49** (2010) 10338]. From the inflection point of the experimental curve (Fig. 5) corresponding to the half-peak potential of the catalytic wave situated at -1.37 V vs Fc+/Fc 15 mV were subtracted to obtain the corrected value of $E^{E}_{1/2} = -1.22$ V. This value was compared with the theoretical value of $E^{T}_{1/2} = -0.68$ V vs. Fc+/Fc reported for 10 mM TFA in acetonitrile given as a reference value in the literature cited above.

From this comparison, **an overpotential of 540 mV can be obtained.**

Current density:

Working electrode (BAS glassy carbon A= 0.0707 cm^2 geometrical surface area) allows for current density (geometrical) calculation at 540 mV overpotential (= half-peak of the catalytic wave, Fig.5):

Catalytic current i_{cat} = 30 µA divided by electrode area leads to: **424 µA cm^{-2} at -1.37 V vs. Fc^+/Fc**

Turnover frequency of the electrocatalyst $Fe_3S_2(CO)_7$dppm:

(pseudo first order constant at high acid concentration excess: k_{obs} = const. = k * conc. of H^+)

Region, where catalytic current i_{cat} does no longer increase with scan rate and H^+ is in excess gives a maximum turnover frequency = k_{obs} (H_2 product elimination is rate limiting step).

Using the equation given in [M. L. Helm, M. P. Stewart, R. M. Bullock, M. R. DuBois, D. L. DuBois, *Science*, **333** (2011) 863] for the maximum i_{cat} value reached at high acid concentrations (asymptotic fit of plot for higher scan rates):

$i_{cat}/ i_p = n /0.446 * \mathrm{sqrt}(R\ T\ k_{obs} / F\ v)$ with n = 2 for hydrogen formation, i_{cat} = catalytic current , i_p = current without acid and v = scan rate in V/s using RT/F =8.3145*298/96485 = 0.0256799

leads tot he following k_{obs} or TOF-values:

TOF in CH_2Cl_2 (130 µA / 27 µA = i_{cat}/i_p at 0.1V/s) = 4.5 s^{-1} = 16200 h^{-1} or 270 min^{-1} (taking the maximum current at more negative wave - see Fig 6!)

TOF in CH_3CN (81.2 µA /27.9 µA = i_{cat}/i_p = 2.911 from asymptotic maximum value reached at 0.1V/s scan rate above 60mM TFA) = ... 1.64 s^{-1} = 5904 h^{-1} or about TOF = 100 min^{-1}

Also a simplified equation for an estimation of the TOF-values can be applied [W. T. Eckenhoff, W. R. McNamara, P. Du, R. Eisenberg, *Biochim. Biophys. Acta* 2013, 1827 (2013) 958]:
k_{obs} = TOF = v * (i_{cat} / 0,72 * i_p)^2 (scan rate v in V/s; 298 K)

Simulations of Electroatalytic Processes

Current-voltage curves measured with the hydrogenase model compound **1** ($Fe_3S_2(CO)_7$dppm) in acetonitrile and dichloromethane in the absence and presence of various amounts of trifluoroacetic acid were calculated and plotted using the digital simulation software package DigiSim (version 3.05).

In reasonable agreement with the experimental results obtained, a C**ECE**C-type reaction mechanism with subsequent chemical steps (C) and electron transfer steps (E) could be assumed as a plausible scenario for modelling the electrocatalytic response of **1**.

<u>The individual steps simulated using DigiSim were as follows:</u>

C: **1** + H^+ = **1H**$^+$ (initial protonation of the catalyst in the presence of added acid)

E: **1H**$^+$ + e^- = **1H** (uptake of the first electron)

C: **1H** + H^+ = **1HH**$^+$ (second protonation step)

E: **1HH**$^+$ + e^- = **1H2** (second reduction step)

C: **1H2** = H2 + **1** (slow release of hydrogen and catalyst regeneration: rate limiting process)

Step outside the catalytic cycle:

H2 = irreversible (gaseous hydrogen evolving and leaving the system)

Some of the calculated contributions to the catalytic currents under different reaction conditions (solvent, TFA concentrations added) together with additional experimental results for low acid concentration are given in Figures S11 – S14 below:

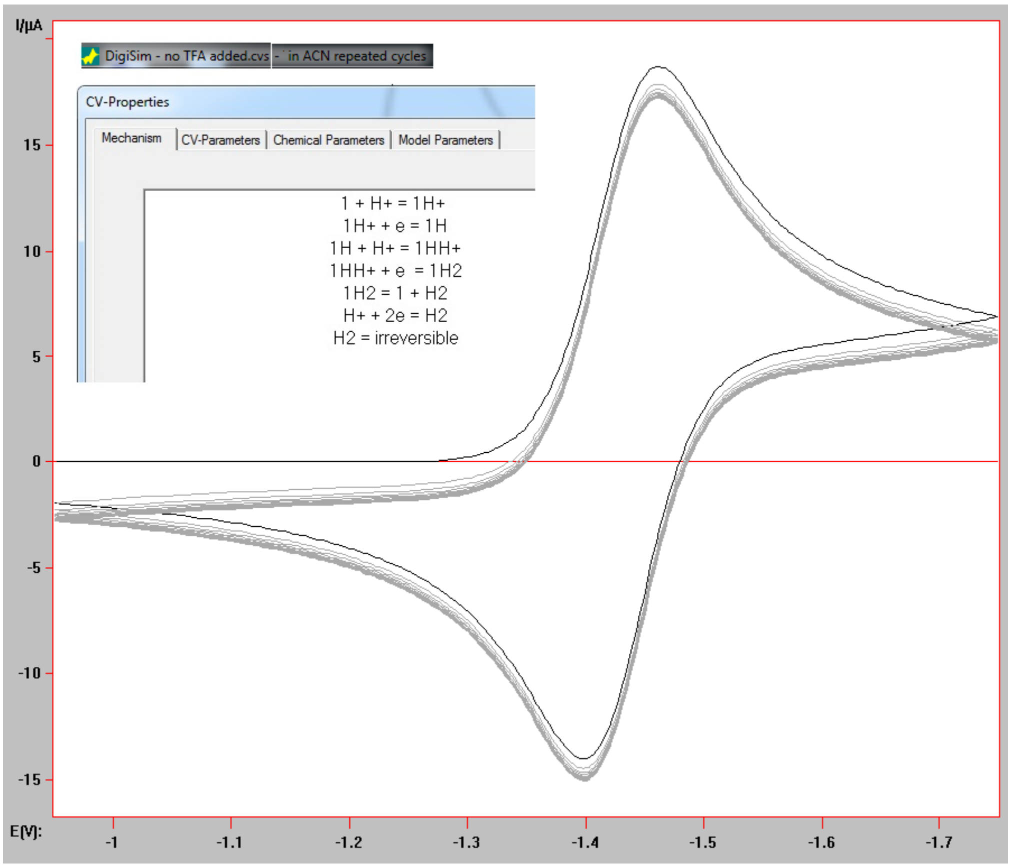

Figure S11. Simulated CVs for a 1.0 mM solution of monoprotonated complex $\mathbf{1H^+}$ in acetonitrile in the absence of excess acid (several repeated scans are shown, scan rate 0.1 V s^{-1}).

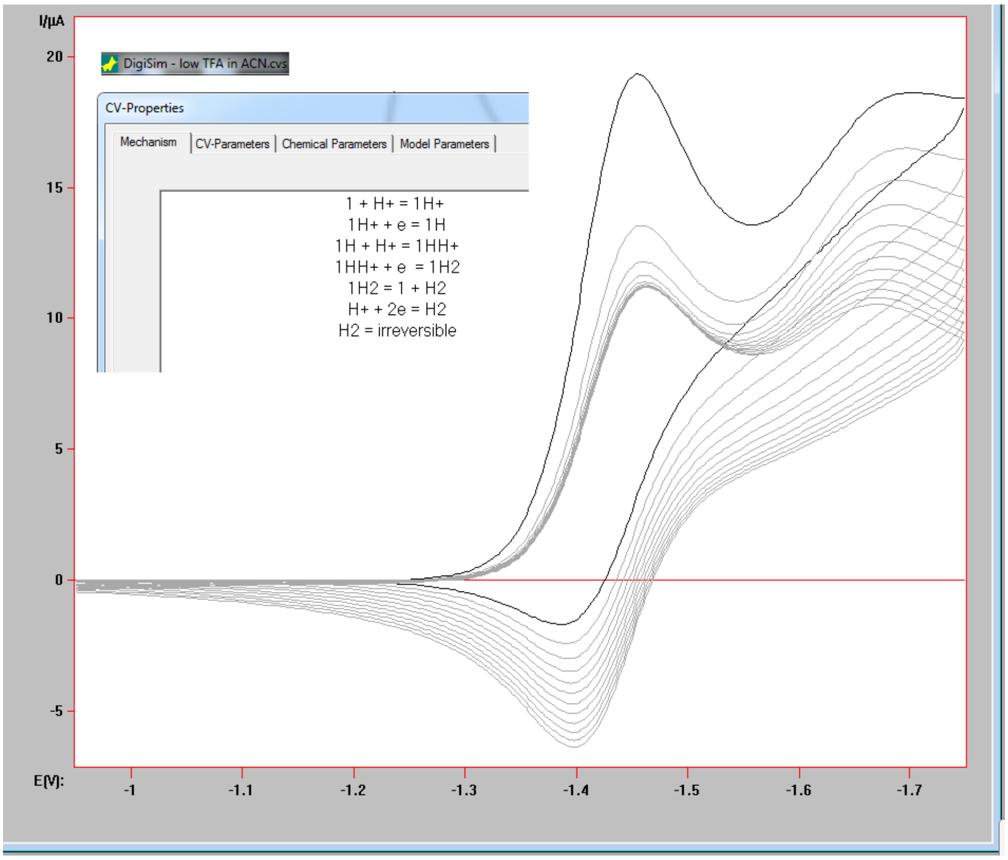

Figure S12. Simulated CVs for a 1 mM solution of monoprotonated complex $\mathbf{1H^+}$ in acetonitrile in the presence of an excess of acid (20 mM TFA). Conditions otherwise as in Fig. S11

**source program: DigiSim for Windows 95**<br>**program version: 3.05**<br>file type: CV<br><br>**charge transfer reactions:**<br>reaction[1]: 1H+ + e = 1H<br>reaction[2]: 1HH+ + e = 1H2<br>reaction[3]: H+ + 2e = H2<br><br>**homogeneous chemical reactions:**<br>reaction[1]: 1 + H+ = 1H+<br>reaction[2]: 1H + H+ = 1HH+<br>reaction[3]: 1H2 = 1 + H2<br>reaction[4]: H2 = irreversible<br><br>experimental parameters:<br>Estart (V): -0.95<br>Eswitch (V): -1.75<br>Eend (V): -0.95<br>v (V/s): 0.1<br>temperature (K): 298.2<br>Ru (Ohms): 0<br>Cdl (F): 0<br>cycles: 12<br>electrode geometry: planar<br>area (cm2): 0.0707<br>diffusion: semi-infinite<br>pre-equilibrium: disabled<br><br>charge transfer parameters:<br>E0[1] (V): -1.43<br>alpha[1]: 0.5<br>ks[1] (cm/s): 0.05<br>E0[2] (V): -1.55<br>alpha[2]: 0.5<br>ks[2] (cm/s): 0.0008<br>E0[3] (V): -1.85<br>alpha[3]: 0.5<br>ks[3] (cm/s): 0.1<br><br>chemical reaction parameters:<br>Keq[1]: 1E+010<br>kf[1]: 1000<br>kb[1]: 1E-007<br>Keq[2]: 1E+010<br>kf[2]: 1000<br>kb[2]: 1E-007<br>Keq[3]: 1000<br>kf[3]: 2<br>kb[3]: 0.002<br>Keq[4]: 1E+005<br>kf[4]: 100<br>kb[4]: 0.001	species parameters:<br>Canal[1H+] (M/l): 0.001<br>Cinit[1H+] (M/l): 0.001<br>D[1H+] (cm2/s): 1E-005<br>Canal[1H] (M/l): 0<br>Cinit[1H] (M/l): 0<br>D[1H] (cm2/s): 1E-005<br>Canal[1HH+] (M/l): 0<br>Cinit[1HH+] (M/l): 0<br>D[1HH+] (cm2/s): 1E-005<br>Canal[1H2] (M/l): 0<br>Cinit[1H2] (M/l): 0<br>D[1H2] (cm2/s): 1E-005<br>Canal[H+] (M/l): 0.002<br>Cinit[H+] (M/l): 0.002<br>D[H+] (cm2/s): 1E-005<br>Canal[H2] (M/l): 0<br>Cinit[H2] (M/l): 0<br>D[H2] (cm2/s): 1E-005<br>Canal[1] (M/l): 0<br>Cinit[1] (M/l): 0<br>D[1] (cm2/s): 1E-005<br>Canal[irreversible] (M/l): 0<br>Cinit[irreversible] (M/l): 0<br>D[irreversible] (cm2/s): 1E-005<br><br>model parameters:<br>expanding space factor: 0.5<br>potential step (V): 0.0025<br>iterations: 1<br>noise_level (A): 0<br>D/k: 50<br>xmax/sqrt(Dt): 6<br>r0 minimum: 20<br><br>simulation statistics:<br>CPU-time: 0.755<br>maximum number of boxes: 20<br>model diffusion coefficient: 2627.5<br>total number of corrections: 0<br>number of potential steps with corrections: 0<br>Imin: -6.3911E-006<br>Imax: 1.931E-005<br>Cmax: 0.002

The simulated CVs shown in Fig. S12 represent the gradual consumption of initially 20 mM TFA during electrocatalytic hydrogen formation catalyzed by complex **1**. Compare these simulated data also with the experimental voltammograms shown in Fig. 5 and with the series of CVs recorded with various TFA concentrations from 0.25 mM up to 5mM (Fig. S13).

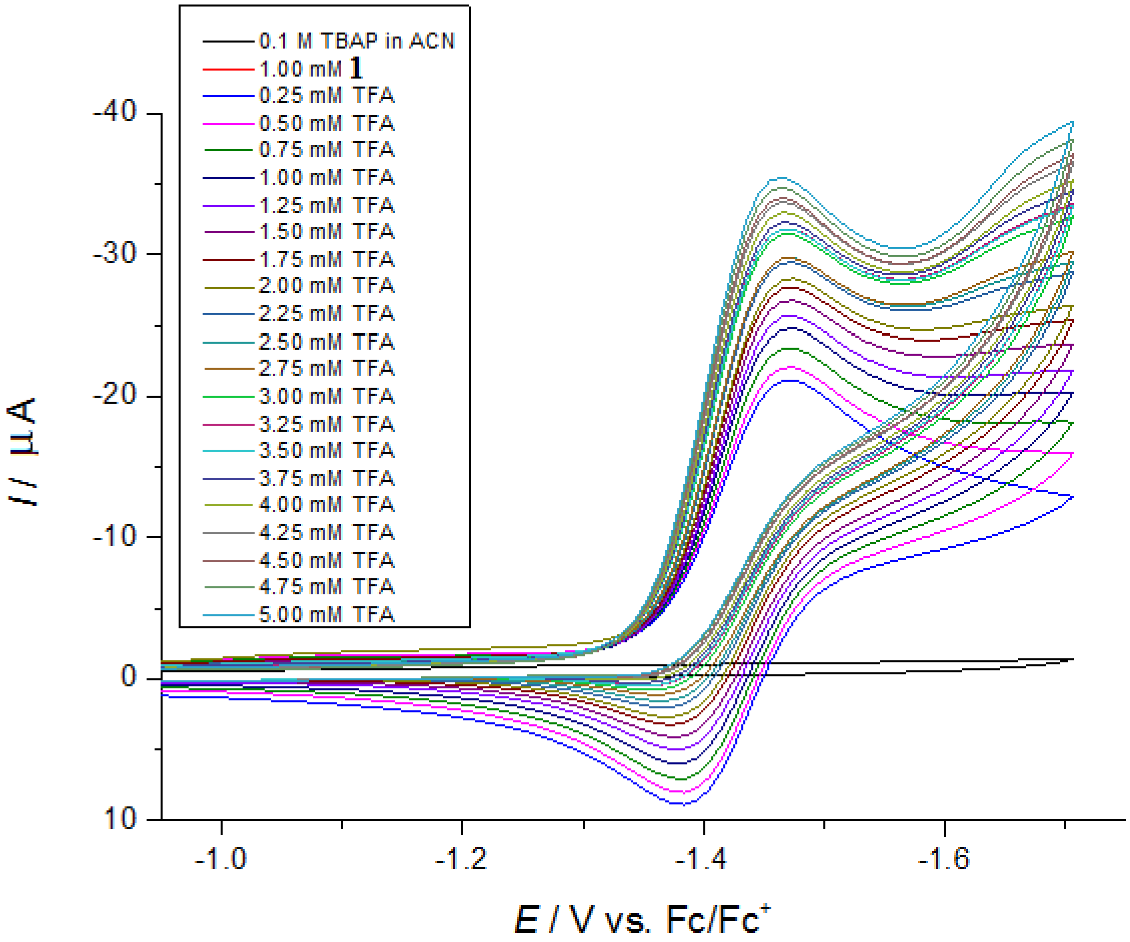

Figure S13. Experimental CVs for a 1 mM solution of complex **1** in acetonitrile in the presence of trifluoroacetic acid at various concentrations.

In Figure S14 below, the simulated contributions to the catalytic currents obtained for complex **1** in dichloromethane are shown in the absence and presence of various amounts of TFA. These data can be compared with the experimental results discussed in the paper (Fig. 6).

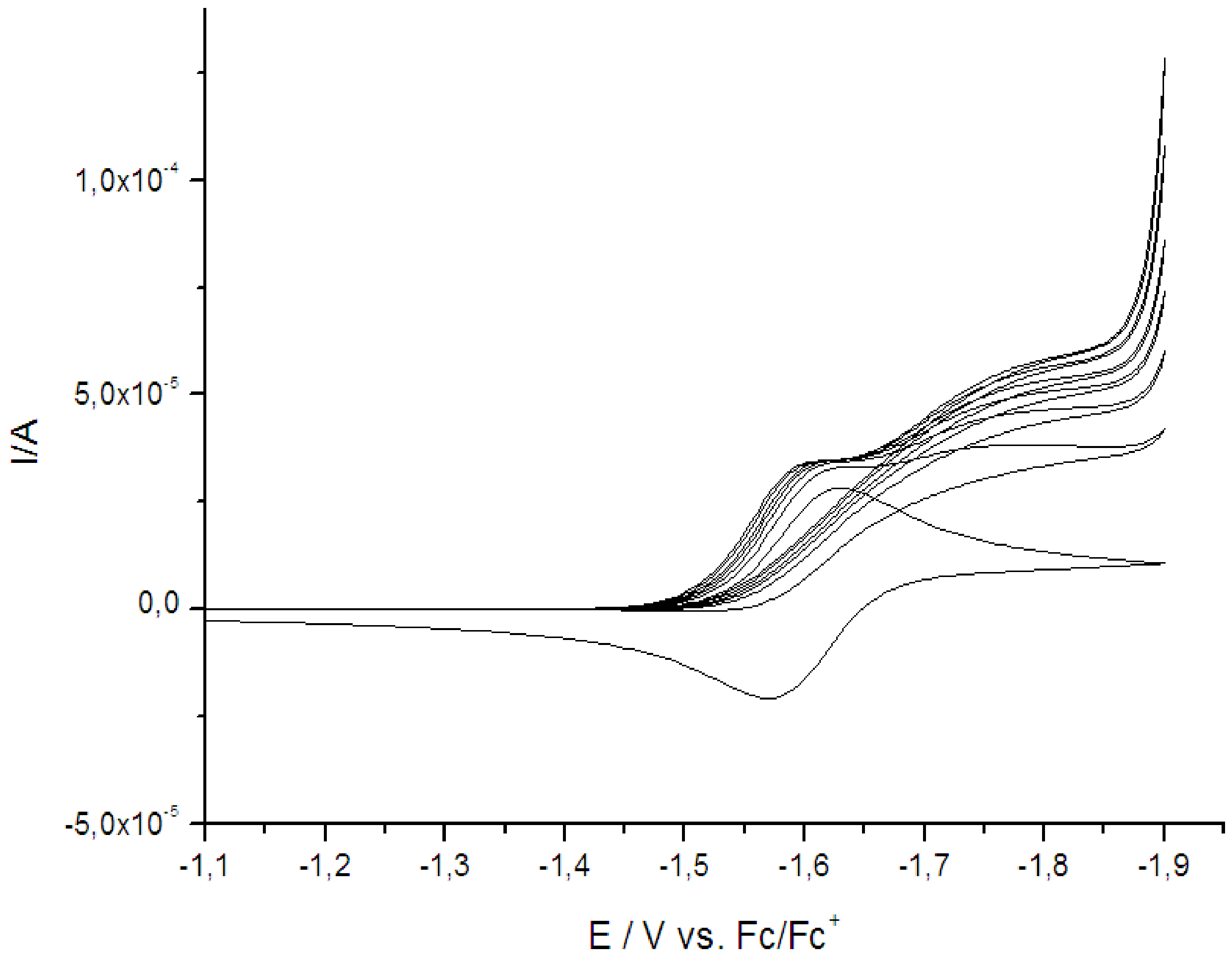

Figure S14. Simulated CVs for a 1.5 mM solution of monoprotonated complex **1H**$^{+}$ in dichloromethane in the absence and presence of excess of trifluoroacetic acid (scan rate 0.1 V s^{-1}). Acid concentrations are 0 mM, 5, 10, 15, 20, 30, and 40 mM, respectively (compare also with Figure S6 in acetonitrile solution and with the experimental data given in Figure 6 of the paper).
